# Supplementary material for: Aberrant FGFR signaling mediates resistance to CDK4/6 inhibitors in ER+ breast cancer
Source: Nat Commun. 2019 Mar 26;10:1373. doi: 10.1038/s41467-019-09068-2 (PMC6435685; doi:10.1038/s41467-019-09068-2)

Supplementary Figure 1. A. Cell viability for all assayed ORFs in presence of fulvestrant (left) and fulvestrant plus ribociclib (right) versus DMSO. **B**. MCF-7 cells stably transduced with constructs encoding eGFP, CDK6, DYRK1B, FGR, GRK6, RAF1, MAP2K6, NTRK1, FRK, FGFR1, HCK, TESK2, NTRK3, PRKAA2, MAPK8, SYK, ROR2 and CRKL were lysed for immunoblot analysis with V5 and tubulin antibodies**. C.** MCF-7 cells expressing eGFP, FGR, GRK6, RAF1, NTRK1, FRK, FGFR1, HCK, TESK2, NTRK3, PRKAA2, MAPK8, SYK, ROR2 and CRKL were treated with fulvestrant/palbociclib or fulvestrant/ribociclib over a dose range for 3 days. Cell proliferation was determined by high-content image counting using ImageXpress Micro Confocal System. Mean ± SD of three replicates are shown (*p<0.05, **p<0.01, p<0.001, Student's t-test).

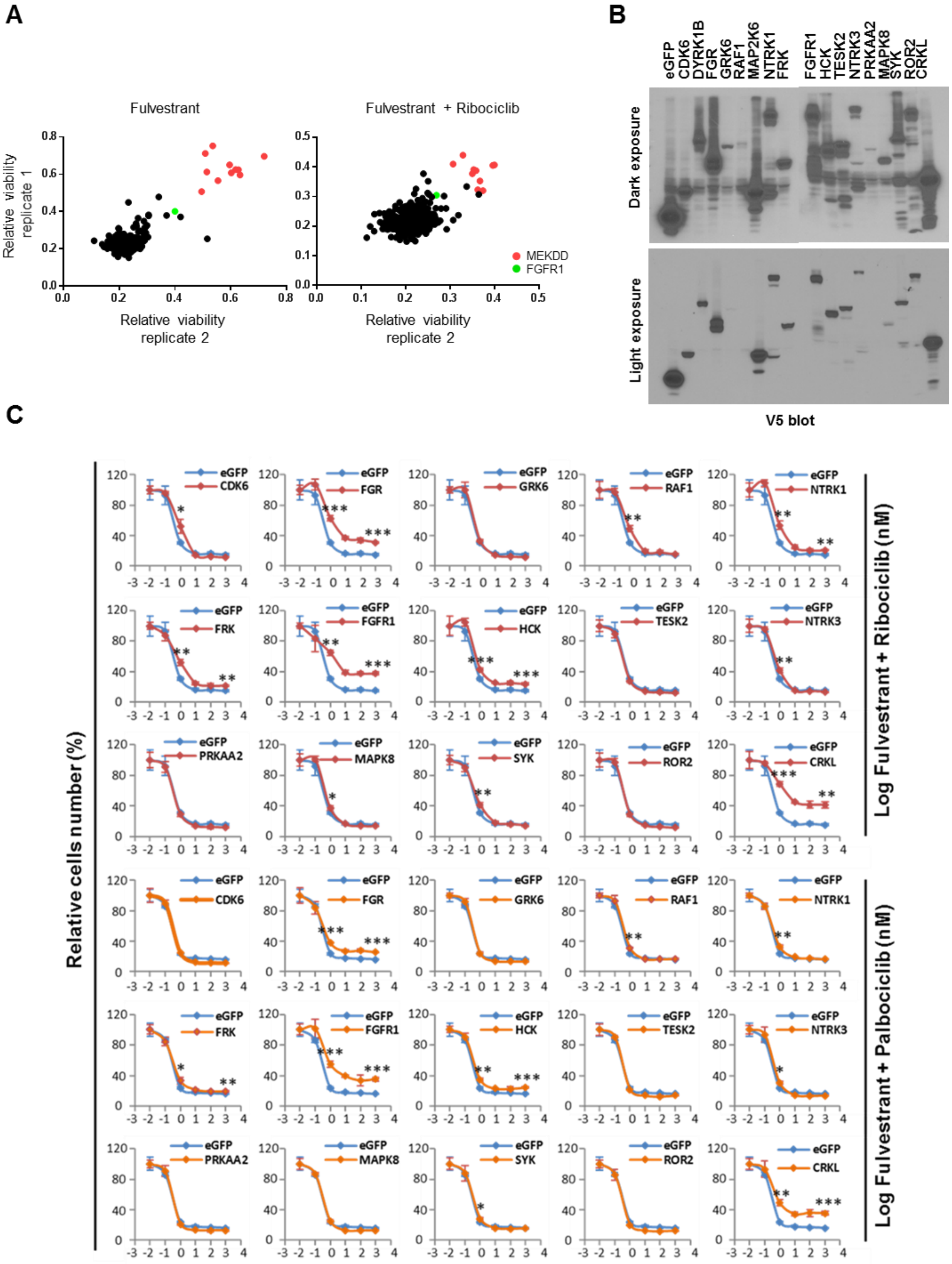
A
Fulvestrant
Fulvestrant + Ribociclib
Relative viability replicate 1
Relative viability replicate 2
MEKDD
FGFR1
B
eGFP
CDK6
DYRK1B
FGR
GRK6
RAF1
MAP2K6
NTRK1
FRK
FGFR1
HCK
TESK2
NTRK3
PRKAA2
MAPK8
SYK
ROR2
CRKL
Dark exposure
Light exposure
V5 blot
C
Relative cells number (%)
Log Fulvestrant + Ribociclib (nM)
Log Fulvestrant + Palbociclib (nM)

Supplementary Figure 2. A. MCF-7^{FGFR1} xenografts were established in ovariectomized athymic mice implanted with a s.c. 14-day release, 0.17-mg 17β-estradiol pellet. Once tumors reached ≥200 mm3, mice were randomized to treatment with vehicle, lucitanib (10 mg/kg/day), fulvestrant (5 mg/week)/lucitanib, or fulvestrant/lucitanib/palbociclib (30 mg/kg/day) for 5-6 weeks. Each data point represents the mean tumor volume in $mm^3 \pm$ SEM (n=8 per arm, ****$p<0.0001$ vs. single drug arms; Student's *t*-test). The table on the top-right shown the volume fold change relative to vehicle. **B.** MCF-7^{eGFP} and MCF-7^{FGFR1} tumors were harvested at the end of treatment. FFPE tumor sections were prepared and subjected to IHC with ERα antibody as described in Methods. The percent of ERα+ tumor cells and their staining intensity were assessed by an expert breast pathologist (P.E.G.) to generate an H-score (HS). **C.** Weight of athymic mice bearing MCF-7^{eGFP} or MCF-7^{FGFR1} mice during treatment as in (A) for a total of 5 and 6 weeks, respectively. Each data point represents mean weight in grams ± SD.

A

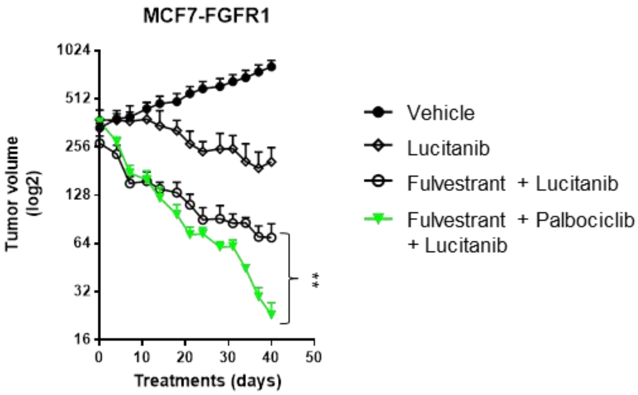

Volume fold change relative to vehicle

	MCF-7^{eGFP}	MCF-7^{FGFR1}
Fulvestrant	-65%	-60%
Lucitanib	-	-65%
Fulvestrant + Lucitanib	-	-90%
Fulvestrant + Palbociclib	-88%	-80%
Fulvestrant + Palbociclib + Lucitanib	-89%	-97%

B

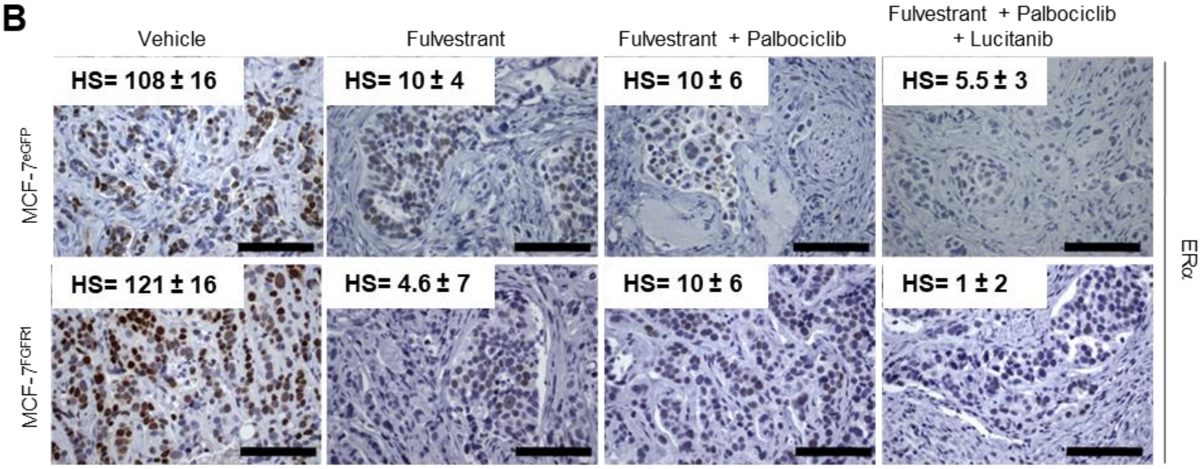

C

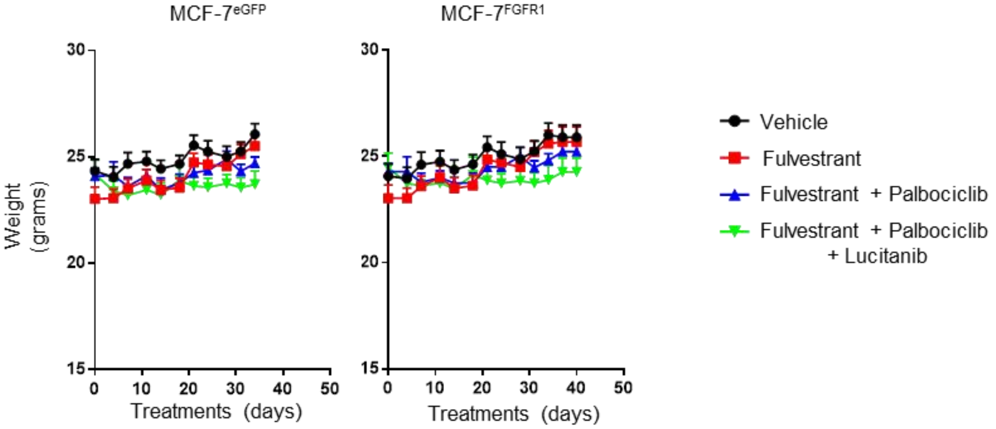

Supplementary Figure 3. A. CAMA1, HCC1500, MDA-MB-134 and MCF-7 were treated with 0-2 μM of fulvestrant/ribociclib or fulvestrant/palbociclib for 3 days. Cell proliferation was determined by high-content image counting using ImageXpress Micro Confocal System. Mean cell number ± SD of three replicates are shown (**p<0.01, Student's t-test). **B.** Average of the fold change in 2D growth with increasing combination doses of fulvestrant and palbociclib (0 to 1000 nM) in absence (left) or in presence (right) of 1 μM lucitanib relative to untreated controls from three independent experiments. A combination Index <1 represents synergism, equal to 1 represents an additive effect, and >1, antagonism. **C.** MDA-MB-134 cells were treated with vehicle (DMSO) or the indicated inhibitors (each at 1 μM) in FGF2-containing media. Cell media and inhibitors were replenished every 3 days. After 21 days, plates were washed and stained with crystal violet; imaging intensity was quantified by spectrophotometric detection. Quantification of the integrated intensity values as fold change relative to vehicle-treated controls are shown (****p<0.0001 vs. controls, Student's t-test). **D.** MDA-MB-134 cells were treated as in (**C**) for 6 h. Cell lysates were prepared and subjected to immunoblot analysis with the indicated antibodies.

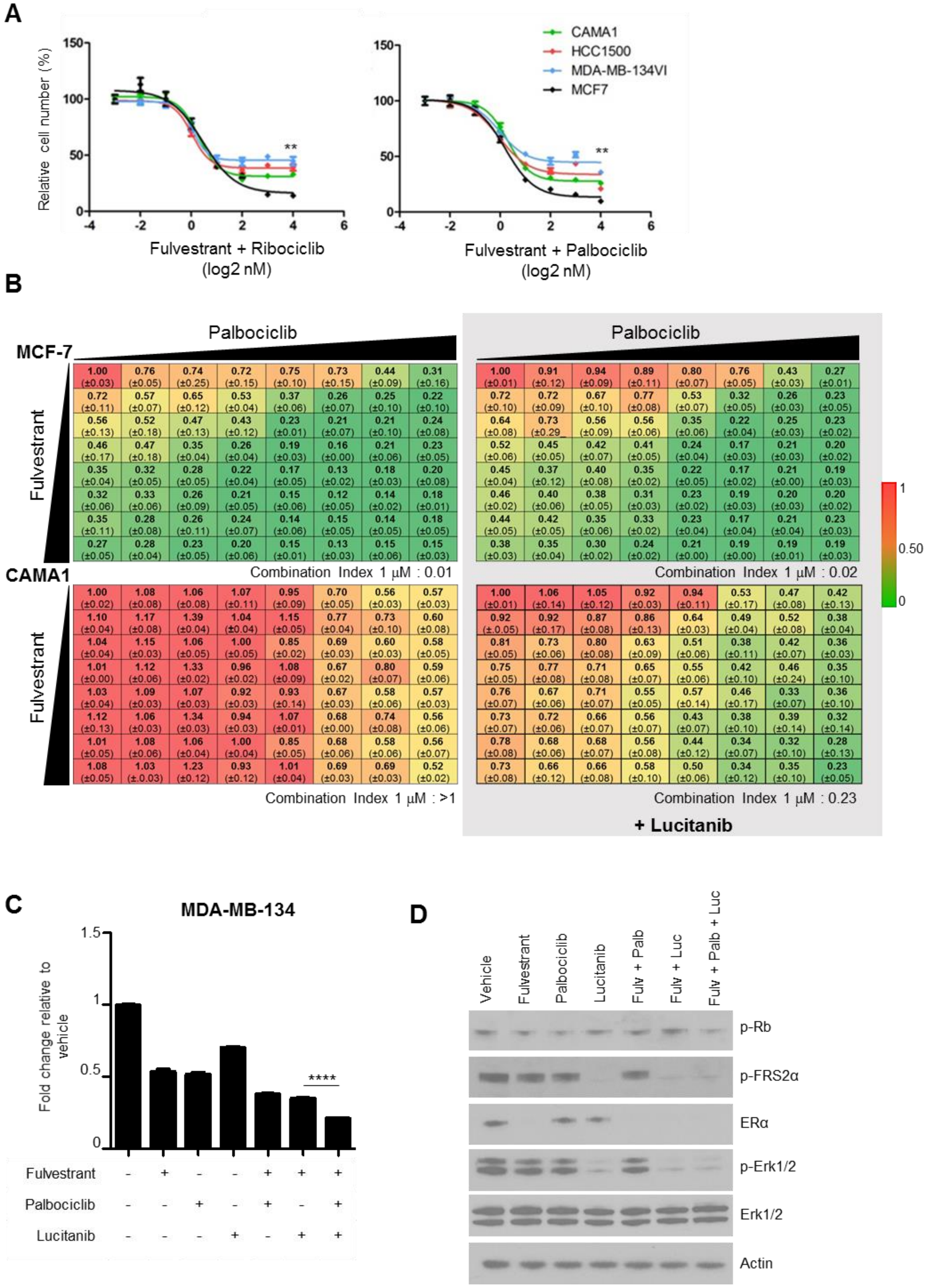
A
Relative cell number (%)
Fulvestrant + Ribociclib (log2 nM)
Fulvestrant + Palbociclib (log2 nM)
CAMA1
HCC1500
MDA-MB-134VI
MCF7
**
B
MCF-7
CAMA1
Palbociclib
Fulvestrant
Combination Index 1 µM : 0.01
Combination Index 1 µM : 0.02
Combination Index 1 µM : >1
Combination Index 1 µM : 0.23
+ Lucitanib
C
MDA-MB-134
Fold change relative to vehicle
****
Fulvestrant
Palbociclib
Lucitanib
D
Vehicle
Fulvestrant
Palbociclib
Lucitanib
Fulv + Palb
Fulv + Luc
Fulv + Palb + Luc
p-Rb
p-FRS2α
ERα
p-Erk1/2
Erk1/2
Actin

Supplementary Figure 4. CAMA1 cells plated in full media containing 10% FBS and FGF2 were transfected with two independent FGFR1 or control siRNAs and treated with vehicle, 1 μM fulvestrant or 1 μM palbociclib or their combination. Four days later, cells were harvested and counted using a Coulter Counter. Each bar represents the fold change relative to vehicle-treated controls. (*p<0.05, **p<0.01 vs. controls, Student's t-test)

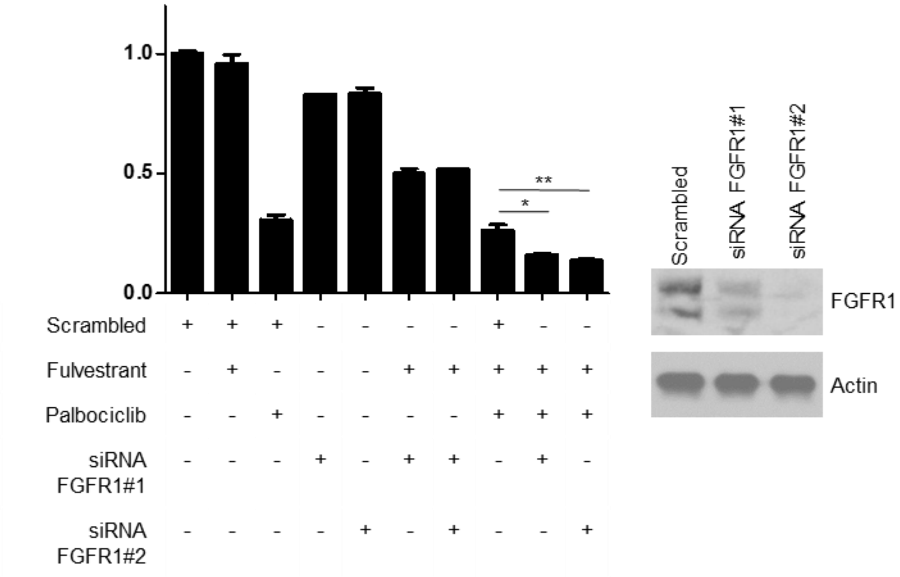

Supplementary Figure 5. CAMA1 and MDA-MB-134 cells were treated with vehicle (DMSO) or the indicated inhibitors (each at 1 μM, except for abemaciclib at 0.5 μM) in FGF2-containing media. Cell media and inhibitors were replenished every 3 days. After 14 days for CAMA1 and 21 days for MDA-MB-134 cells, plates were washed and stained with crystal violet; imaging intensity was quantified by spectrophotometric detection. Fold change of the integrated intensity values relative to vehicle-treated controls are shown (****p<0.0001 vs. controls, Student's t-test).

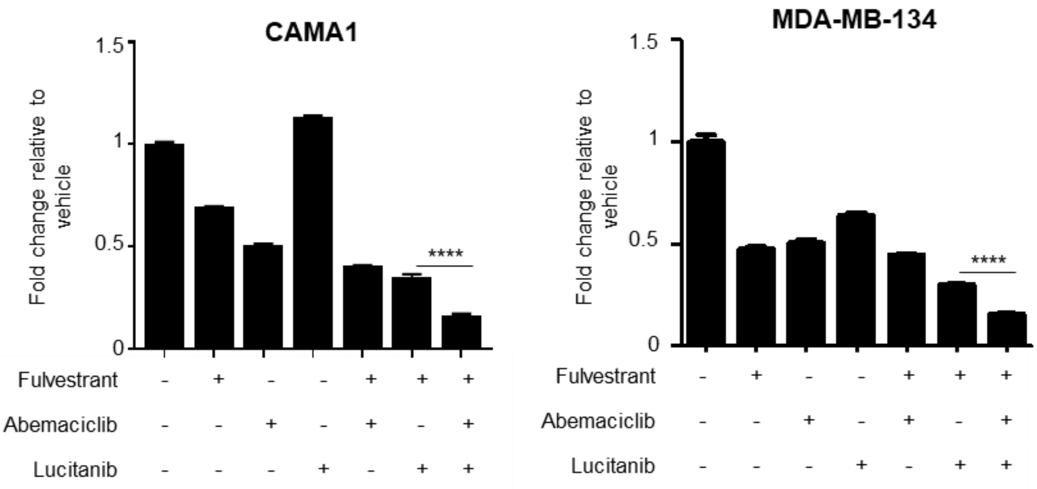

Supplementary Figure 6. A-B. CAMA1 cells plated in full media containing FGF2 were treated with vehicle, fulvestrant, palbociclib, selumetinib, lucitanib (each at 1μM) alone or in combination as indicated. Drugs and media were replenished every 3 days. Fourteen days later, monolayers were stained with crystal violet and analyzed as described in Methods. Representative images (**A**) and quantification of the integrated intensity values as fold change relative to vehicle-treated controls (**B**) are shown (****p<0.0001 vs. controls, Student's t-test). **C**. CAMA1 cells were treated as in (**A**) for 6 h. Cell lysates were prepared and subjected to immunoblot analysis with the indicated antibodies.

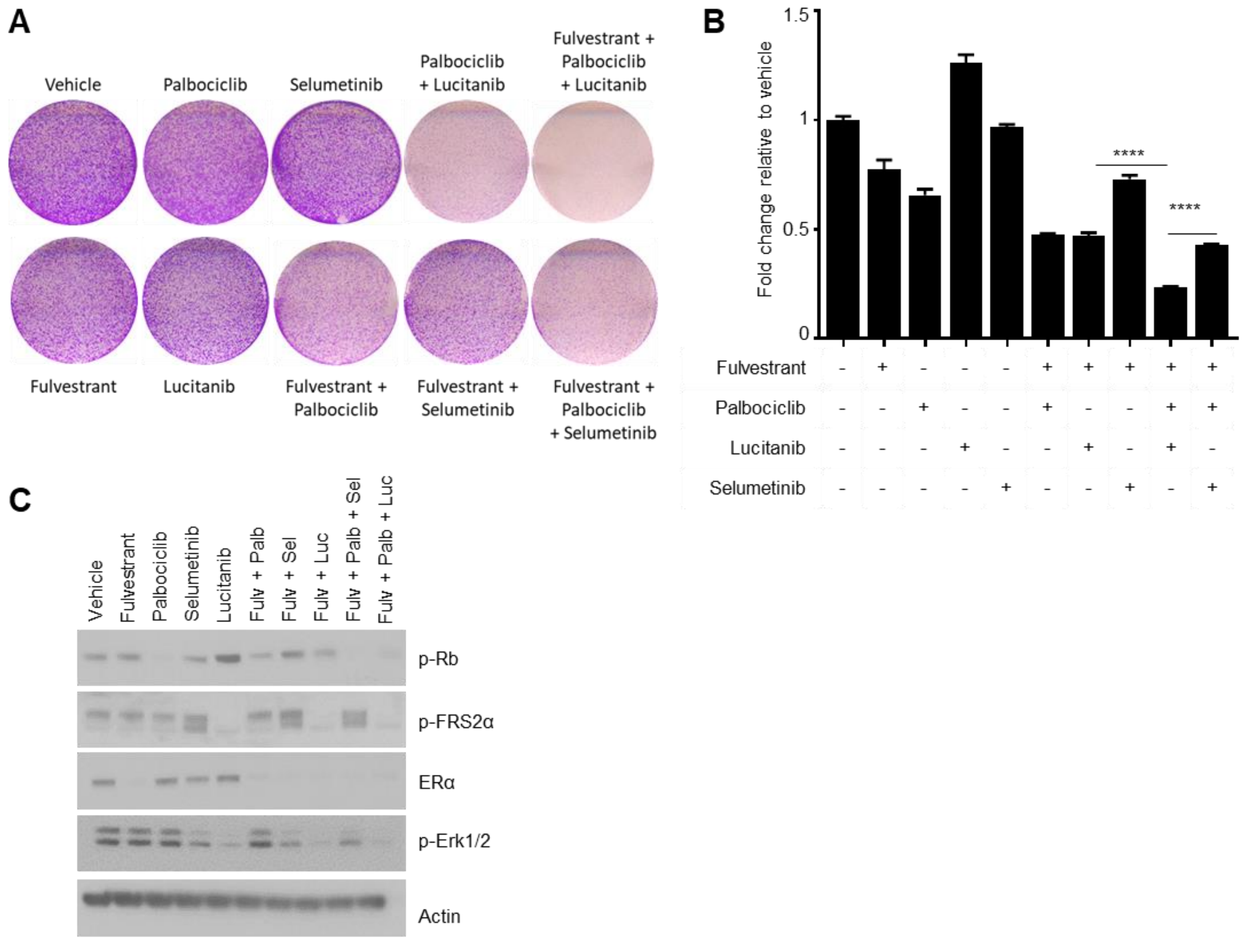

Supplementary Figure 7. A. Tile plot of ER+, *FGFR1*-amplified/overexpressing vs ER+, non-*FGFR1*-amplified/overexpressing breast cancers in TGCA (Cell 2015). The tile plot showed that breast cancers with FGFR1 overexpression and/or amplification have high levels of CCND1 by mRNA and/or RPPA. **B-C.** Scatter plot showing higher levels of CCND1 mRNA and and protein in all ER+/*FGFR1*-amplified/overexpressing breast cancers (FGFR1 ALT) with (**B**) or without (**C**) *CCND1* amplification (TGCA, Cell 2015).

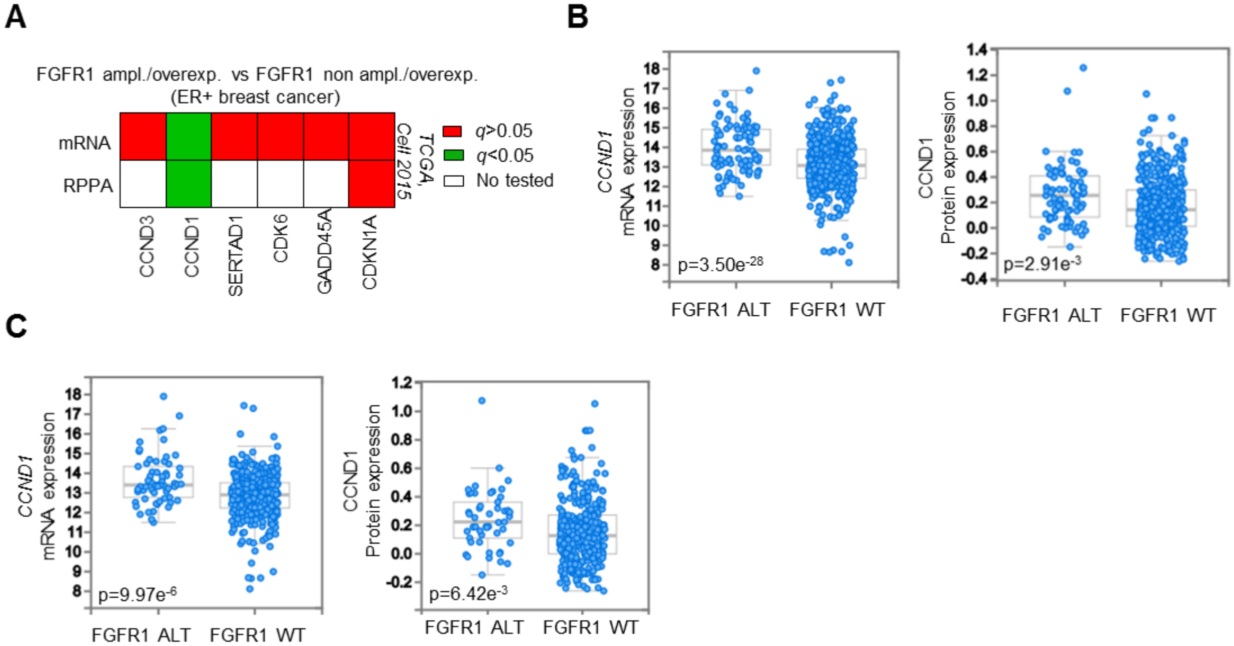

Supplementary Figure 8. A. CAMA1 cells plated in full media containing 10% FBS and FGF2 were transfected with two independent CCND1 or control siRNAs and treated with vehicle or lucitanib as described in Methods. Four days later, cells were harvested and counted using a Coulter Counter. Each bar represents the fold change relative to vehicle-treated controls. **B**. Cyclin D1 knockdown was confirmed by immunoblot analysis of cell lysates from plates treated as in (**A**) for 4 days. **C.** CAMA1 cells in FGF2 containing DCC-FBS were transfected with FGFR1 siRNAs, CCND1 siRNAs or control siRNAs as described in Methods. Seven days later, monolayers were harvested and cell counts determined using a Coulter Counter. Each bar represents the fold change relative to vehicle-treated controls (****p<0.0001 vs. control siRNA, Student's t-test). **D**. FGFR1 and Cyclin D1 knockdown were confirmed by immunoblot analysis of lysates of cells treated as in (**C**) 3 days after transfection.

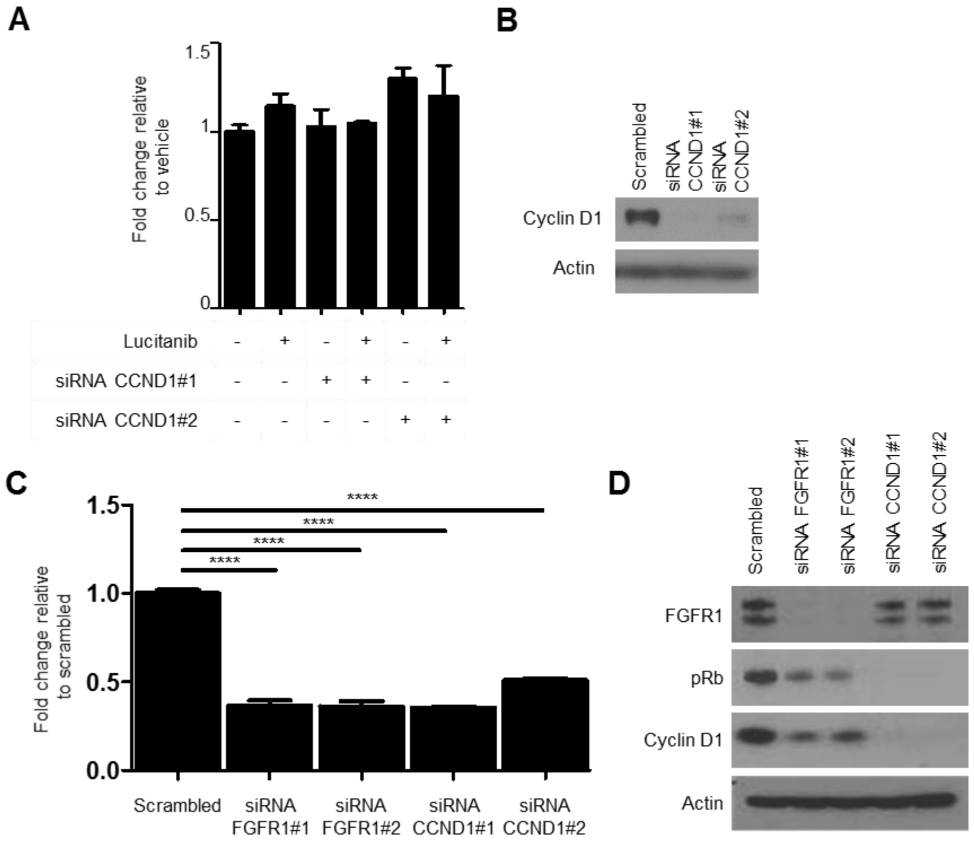

Supplementary Figure 9. A. CAMA1 cells plated in full media containing FGF2 were treated with vehicle, 1 μM fulvestrant, 1 μM palbociclib, 0.01 μM erdafitinib, each alone or in combination as indicated. Drugs and media were replenished every 3 days. Fourteen days later, monolayers were stained with crystal violet and analyzed as described in Methods. Quantification of the intensity values as fold change relative to vehicle-treated controls are shown (****p<0.0001 vs. controls, Student's t-test). **B.** CAMA1 cells were treated with vehicle, 1 μM fulvestrant, 1 μM palbociclib, 0.25 μM erdafitinib for 6 h. Cell lysates were prepared and subjected to immunoblot analysis with the indicated antibodies.

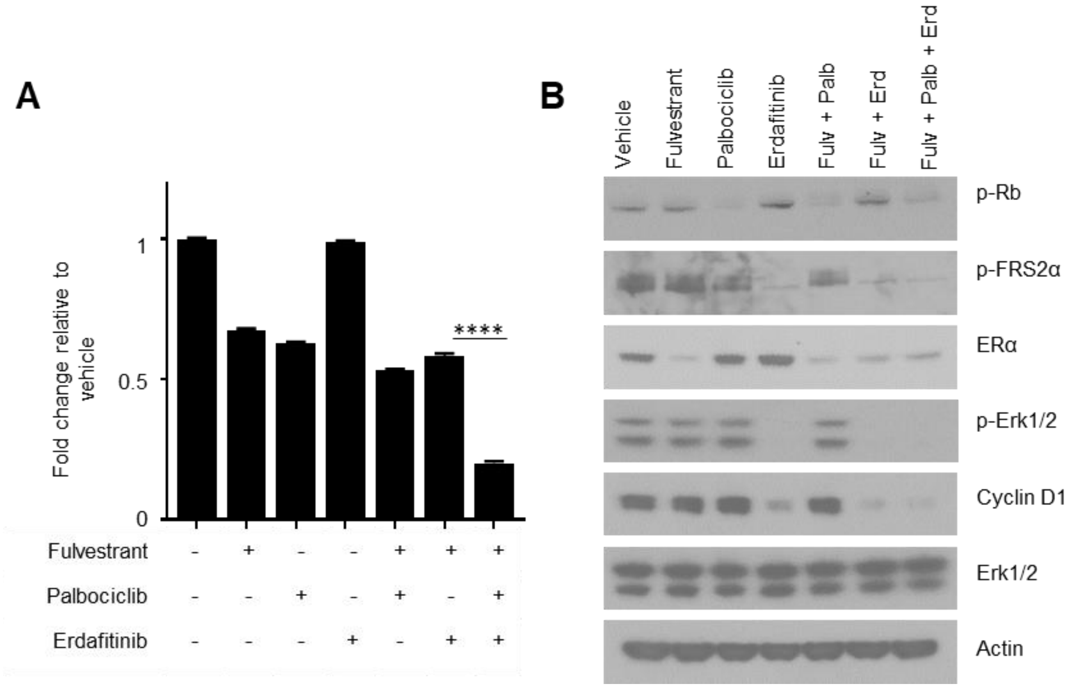

Supplementary Figure 10. TM00368 tumors were harvested after one week of treatment with the indicated inhibitors. FFPE tumor sections subjected to IHC to detect the Ki67 positive cells. The dot plots represents the percentage of Ki67 positive tumor cells (*p<0.05, Student's t-test).

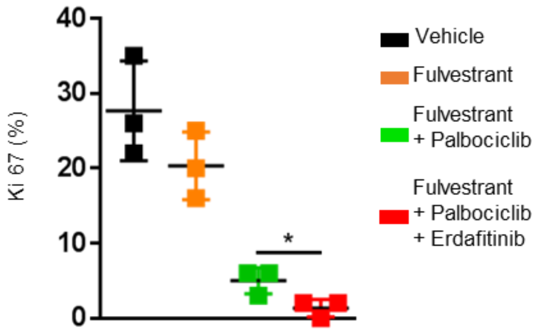

Supplementary Figure 11. TM00368 PDX were established in ovariectomized SCID/beige mice as described in the Methods. Once tumors reached ≥200 mm^3, mice were randomized to treatment for 1 week (n=3 per group). The percent of Ki67 tumor cells was measured by FACS as described in Methods. Gates in the dot plots represent the percent of of Ki67+ tumor cells (*p<0.05, Student's t-test).

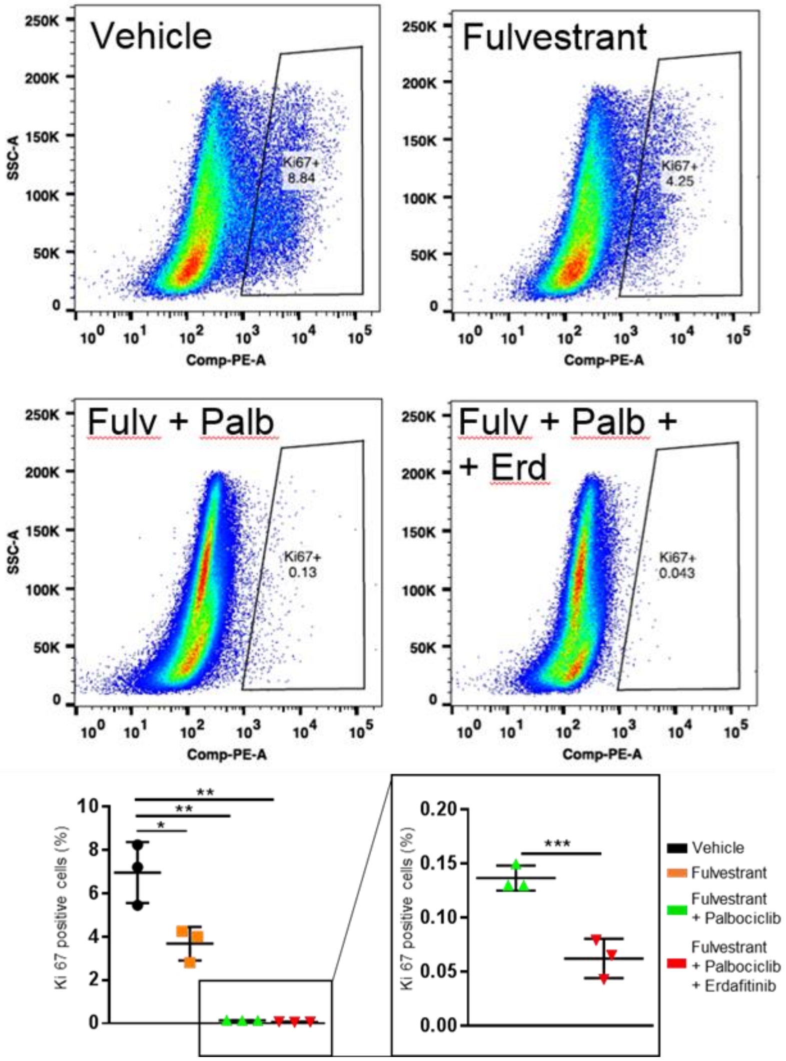

Supplementary Figure 12. A-B. TM00368 tumors were harvested after 3 weeks of treatment with the indicated inhibitors. FFPE tumor sections were subjected to IHC with S807/811 pRb and cyclin D1 antibodies as described in Methods. The percent of p-Rb+ and cyclin D1+ tumor cells and their staining intensity were assessed by an expert breast pathologist (P.G.E.) blinded to treatment arm to generate an H-score. Total p-Rb (D) and cyclin D1 (E) H-scores are shown (**p<0.01, Student's t-test). **C-D**. Representative FFPE sections from tumors treated with fulvestrant or erdafitinib subjected to IHC with ERα and p-FGFR1 antibodies.

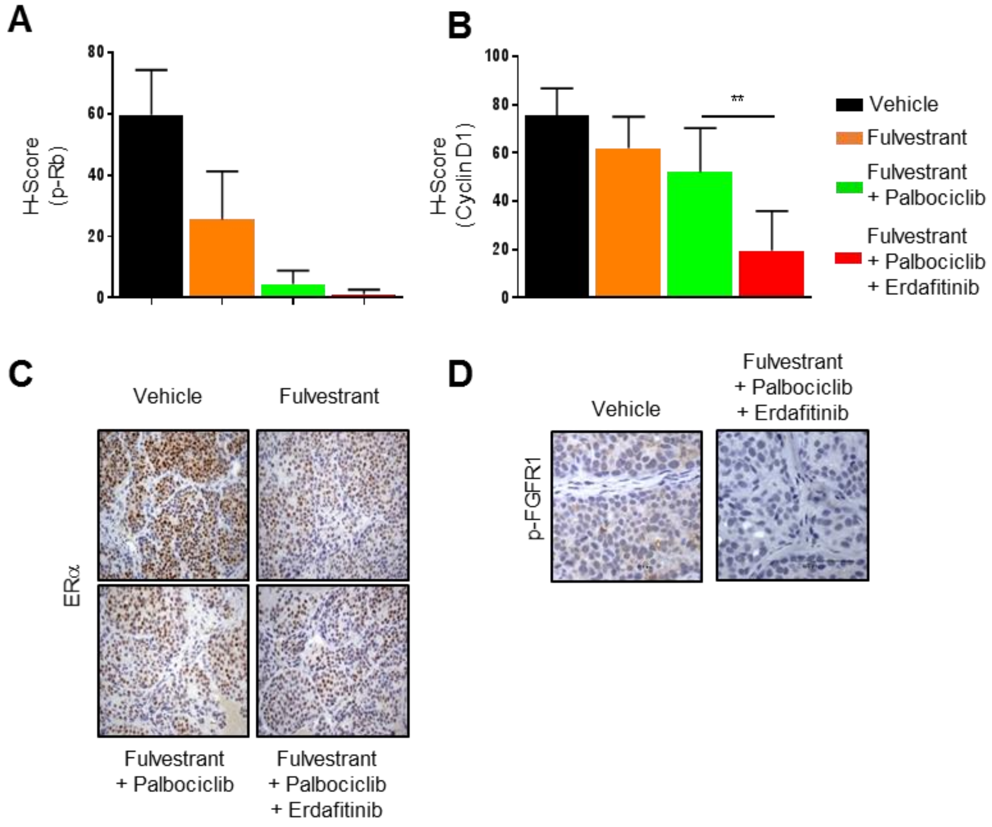

Supplementary Figure 13. A. TM00368 tumors were harvested at the end of treatment with the indicated inhibitors. FFPE tumor sections were subjected to TUNNEL analysis. The percent of tunnel positive tumor cells was assessed by an expert breast pathologist (P.G.E.) blinded to treatment arm. The percentage of the TUNNEL positive tumor cells is shown. **B**. Weight of SCID/beige mice implanted with TM00368 ER+/F*GFR1*-amplified tumors during treatment with the indicated drugs for 3 weeks. Each data point represents mean mouse weight in grams ± SD (n=8per arm).

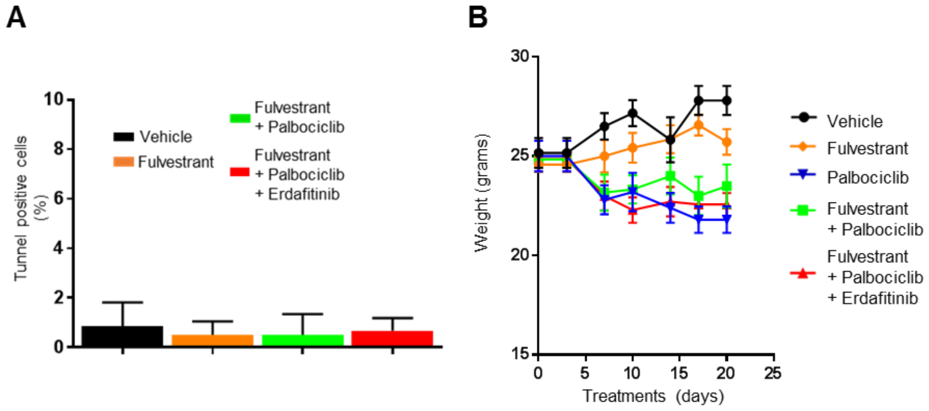

Supplementary Figure 14. A. FGFR1 (●) and FGFR2 (Δ) in pre-treatment and post-progression ctDNA in patients treated with letrozole or fulvestrant plus palbociclib. Y-axis indicates the plasma absolute copy number in ctDNA with the following range value: 2.14-2.39 (low level amplification), 2.4-4.0 (moderate), >4.0 (high) (N.D.= no detectable) **B**. MCF-7^{eGFP} and MCF-7^{FGFR2} cells were seeded in 6-well plates in full media supplemented with 2 ng/mL FGF2 and treated with vehicle (DMSO) or 1 μM fulvestrant + 1 μM palbociclib. Drugs and media were replenished every 3 days. After 14 days, monolayers were stained with crystal violet and analyzed as described in Methods. Representative images and quantification of the integrated intensity values as fold change relative to vehicle-treated controls are shown (****p<0.0001 vs. controls, Student's t-test). FGFR2 overexpression was detected by immunoblot analysis.

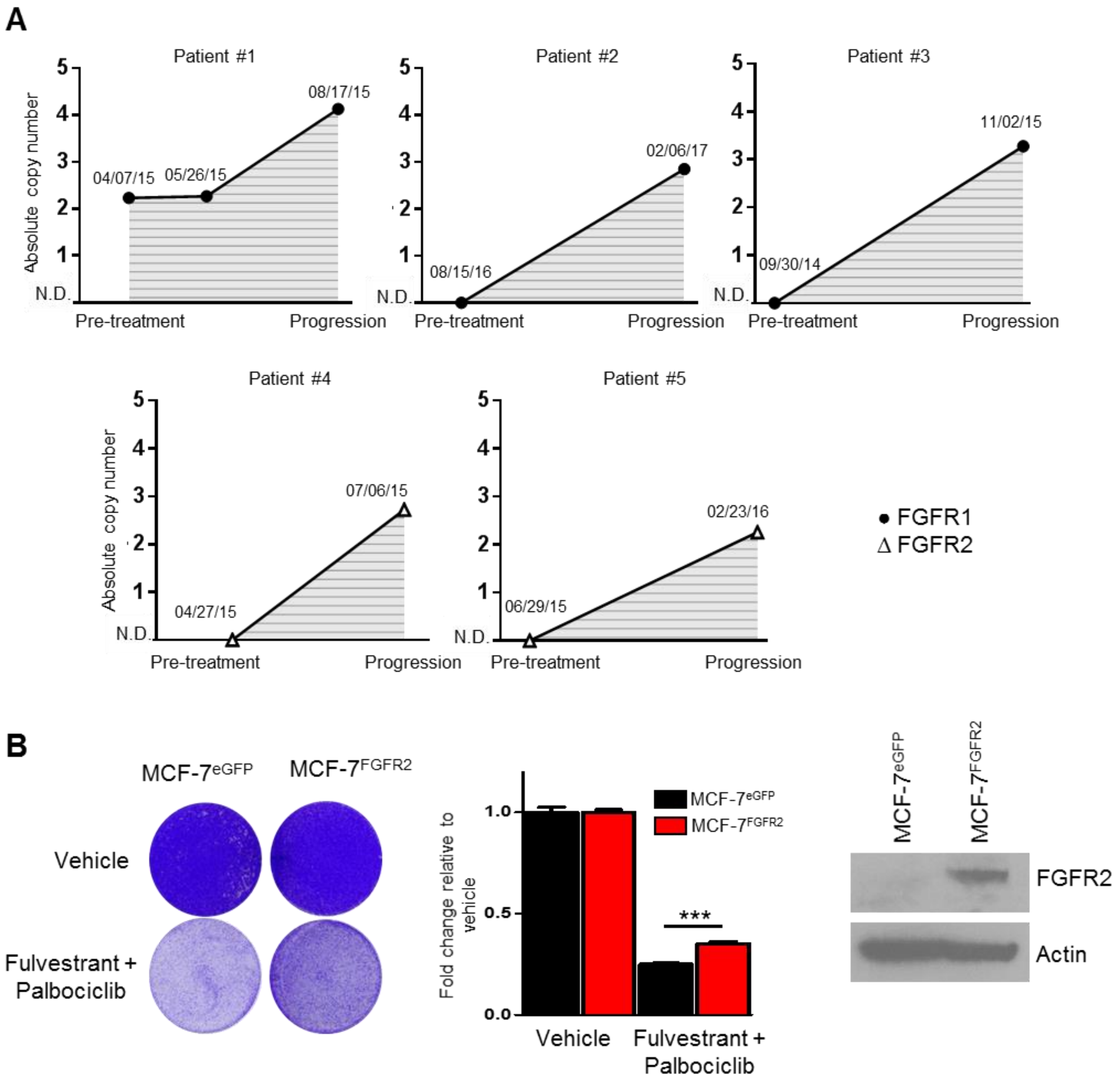
A
Patient #1
Patient #2
Patient #3
Patient #4
Patient #5
Absolute copy number
N.D.
Pre-treatment
Progression
04/07/15
05/26/15
08/17/15
08/15/16
02/06/17
09/30/14
11/02/15
04/27/15
07/06/15
06/29/15
02/23/16
● FGFR1
Δ FGFR2
B
MCF-7eGFP
MCF-7FGFR2
Vehicle
Fulvestrant + Palbociclib
Fold change relative to vehicle
***
FGFR2
Actin

Supplementary Figure 15. Progression free survival plots of patients in MONALEESA-2 as a function of high vs. low *CCNE1* and *CDK2* mRNA levels. Patients treated with letrozole/ribociclib and with high *CCNE1* mRNA but not *CDK2* mRNA in RNA extracted from archival tumor biopsies exhibited a statistically shorter PFS than patients with low *CCNE1* mRNA.

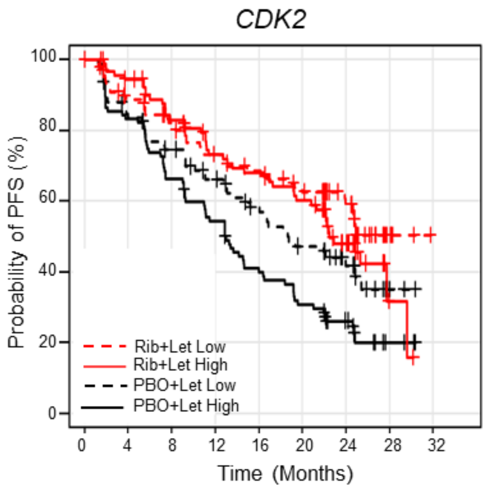

Legend	Group	N	Events	Median PFS (95% CI)	HR (95% CI)	*P*
	Rib + Let CDK2 high	94	45	22,47 (19,42 – 29,6)	0.83 (0.54 – 1,27)	3,9e-01
	Rib + Let CDK2 low	103	40	NR (23.85 – NR)		
	PBO + Let CDK2 high	101	71	12,98 (9,23 – 15,97)	0.65 (0.45 – 0,94)	2,2e-02
	PBO + Let CDK2 low	93	49	18,73 (13,83 – 25,4)		

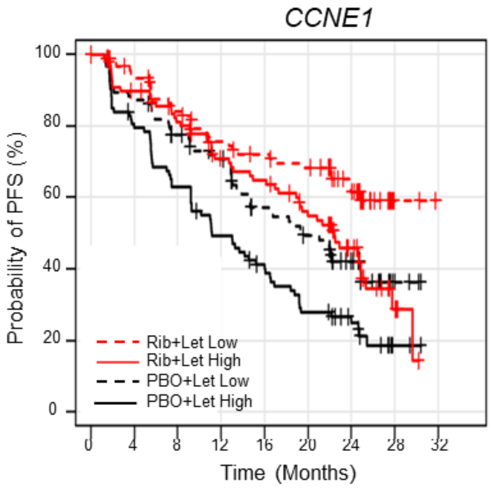

Legend	Group	N	Events	Median PFS (95% CI)	HR (95% CI)	*P*
	Rib + Let CCNE1 high	100	53	22,41 (18,89 – 24,87)	0.58 (0.37 – 0,89)	1,4e-02
	Rib + Let CCNE1 low	97	32	NR (23.92 – NR)		
	PBO + Let CCNE1 high	95	69	11,1 (9,17 – 16,03)	0.59 (0.41 – 0,85)	4,8e-03
	PBO + Let CCNE1 low	99	51	19,55 (14,52 – 24,84)		

Supplementary Table 1.

Hits	Secondary screen at low dose		Secondary screen at high dose	
	Fulvestrant 1nM + Ribociclib 1nM	Fulvestrant 1nM + Palbociclib 1nM	Fulvestrant 1μM + Ribociclib 1μM	Fulvestrant 1μM + Palbociclib 1μM
CDK6	p<0.05	No resistance	No resistance	No resistance
**FGR**	**p<0.001**	**p<0.001**	**p<0.001**	**p<0.001**
GRK6	No resistance	No resistance	No resistance	No resistance
RAF1	p<0.01	p<0.01	No resistance	No resistance
NTRK1	p<0.01	p<0.01	p<0.01	No resistance
**FRK**	**p<0.01**	**p<0.05**	**p<0.01**	**p<0.01**
**FGFR1**	**p<0.01**	**p<0.001**	**p<0.001**	**p<0.001**
**HCK**	**p<0.001**	**p<0.01**	**p<0.001**	**p<0.001**
TESK2	No resistance	No resistance	No resistance	No resistance
NTRK3	p<0.01	p<0.05	No resistance	No resistance
PRKAA2	No resistance	No resistance	No resistance	No resistance
MAPK8	p<0.05	No resistance	No resistance	No resistance
SYK	p<0.01	p<0.05	No resistance	No resistance
ROR2	No resistance	No resistance	No resistance	No resistance
**CRKL**	**p<0.001**	**p<0.01**	**p<0.01**	**p<0.001**

Supplementary Figure 16. Uncropped images of the most relevant immunoblots are shown.

Figure 2

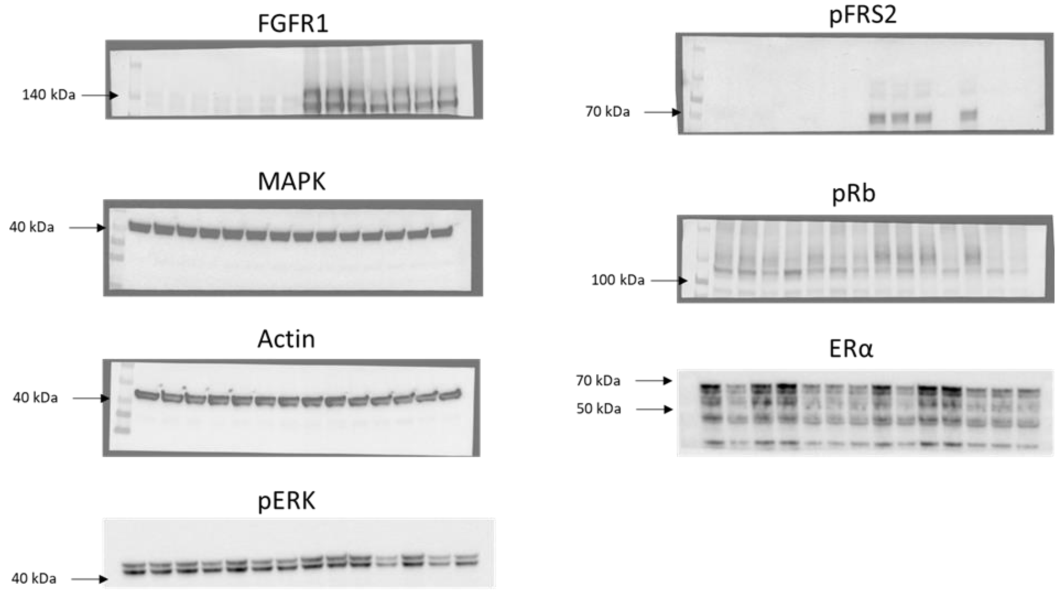

Figure 5C

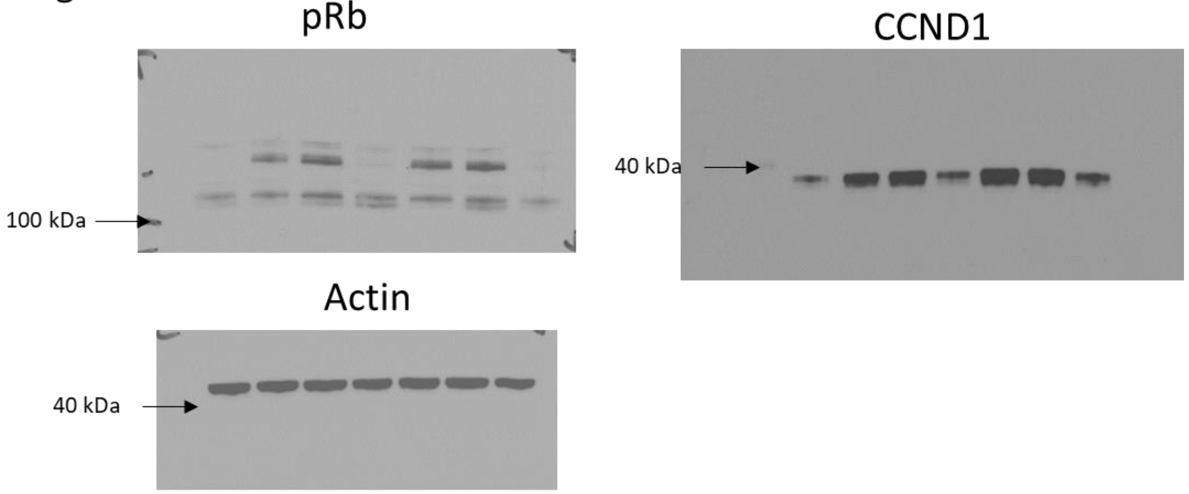

Figure 5E

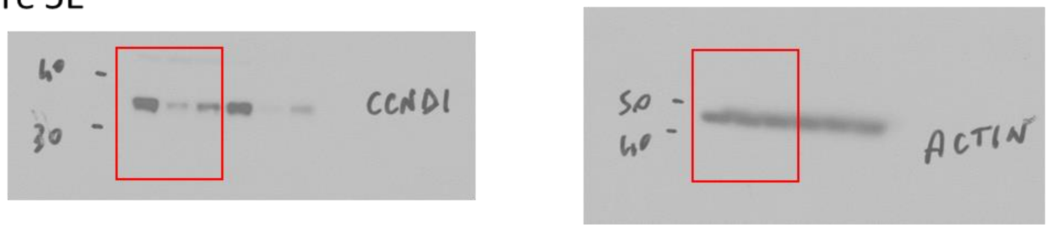

Figure 6G

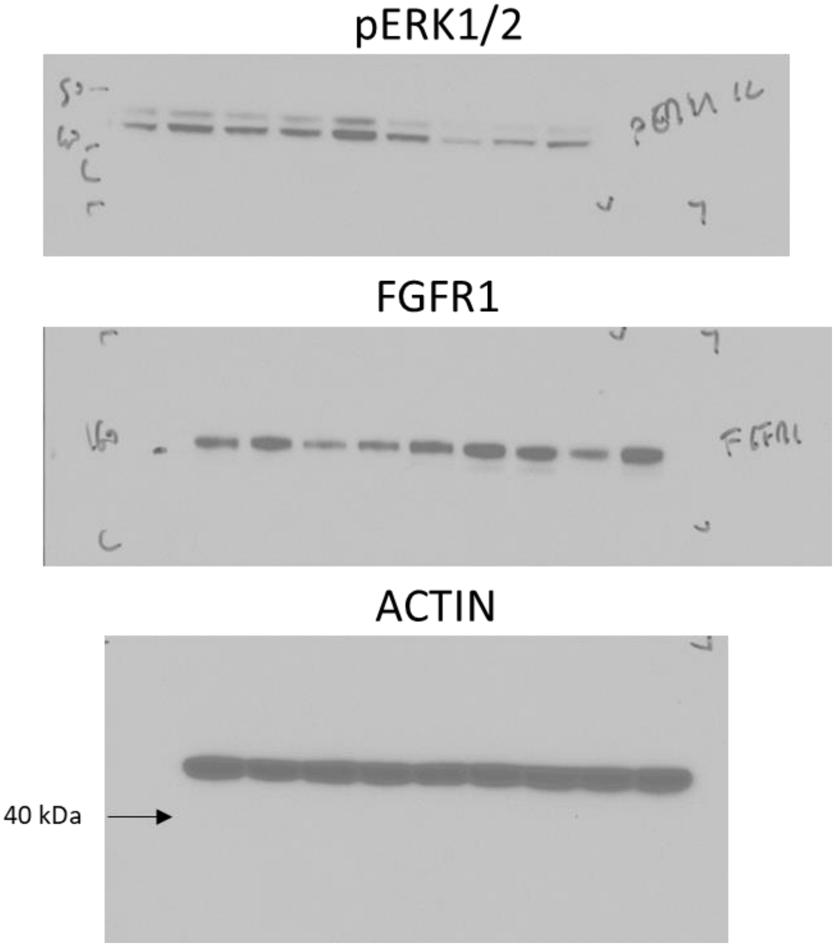

Supplement: Supplementary file 1 — Supplementary Information [file 41467_2019_9068_MOESM1_ESM.pdf]
