# Supplementary material for: Aberrant FGFR signaling mediates resistance to CDK4/6 inhibitors in ER+ breast cancer
Source: Nat Commun. 2019 Mar 26;10:1373. doi: 10.1038/s41467-019-09068-2 (PMC6435685; doi:10.1038/s41467-019-09068-2)
Supplement: Supplementary file 3 — Description of Additional Supplementary Files [file 41467_2019_9068_MOESM3_ESM.docx]

**Description of Supplementary Files**

**File Name:** Supplementary Data 1

**Description:** Nanostring analysis in TM00386 PDX – original data (Figure 6D)

**File Name:** Supplementary Data 2

**Description:** Nanostring analysis in tissue samples obtained from MONALEESA-2 trial – original data (Figure 7C)

**File Name:** Supplementary Data 3

**Description:** ctDNA NGS analysis obtained from ER+/HER2– breast cancer patients treated with palbociclib plus endocrine therapy – original data (Figure 7A)

**File Name:** Supplementary Data 4

**Description:** Original data from the NGS analysis of the ctDNA obtained from MONALEESA-2 trial (Figure 7B)

**File Name:** Supplementary Data 5

**Description:** RNA-seq analysis in Cama1 ER+/HER2- breast cancer cell line – original data (Figure 4)
